# Contemporary treatment patterns and survival of cervical cancer patients in Ethiopia

**DOI:** 10.1186/s12885-021-08817-1

**Published:** 2021-10-13

**Authors:** Biniyam Tefera Deressa, Mathewos Assefa, Ephrem Tafesse, Eva Johanna Kantelhardt, Ivan Soldatovic, Nikola Cihoric, Daniel Rauch, Ahmedin Jemal

**Affiliations:** 1 Adama Hospital Medical College, Adama, Ethiopia; 2 Department of Clinical Oncology, College of Health Sciences, Addis Ababa university, Addis Ababa, Ethiopia; 3 College of Medicine and Health Sciences, University of Gondar, Gondar, Ethiopia; 4 Department of Gynaecology and Institute of Clinical Epidemiology, Martin Luther University, Halle an der Saale, Germany; 5 Institute of Medical Statistics and Informatics, Faculty of Medicine, University of Belgrade, Beograd, Serbia; 6 Department of Radiation Oncology, Inselspital, Bern University Hospital, University of Bern, Bern, Switzerland; 7 Department of Medical Oncology, Inselspital, Bern University Hospital, University of Bern, Bern, Switzerland; 8 Surveillance and Health Equity Science, American Cancer Society, Atlanta, Georgia USA

**Keywords:** Cervical cancer, Overall survival, Tikur Anbessa specialized hospital, Ethiopia

## Abstract

**Background:**

Cervical cancer is the second commonly diagnosed cancer and the second leading cause of cancer death in women in Ethiopia, with rates among the highest worldwide. However, there are limited data on cervical cancer treatment patterns and survival in the country. Herein, we examine treatment patterns and survival of cervical cancer patients treated in Tikur Anbessa Hospital Radiotherapy Center (TAHRC), the only hospital with radiotherapy facility in the country.

**Methods:**

Women with histologically verified cervical cancer who were seen in 2014 (January 1, 2014 to December 31, 2014) at TAHRC were included. Information about clinical characteristics and treatments were extracted from the patients’ medical record files. The information on vital status was obtained from medical chart and through telephone calls.

**Result:**

Among 242 patients included in the study, the median age at diagnosis was 48 years. The median waiting time for radiotherapy was 5.6 months (range 2 to 9 months). Stage migration occurred in 13% of patients while waiting for radiotherapy. Consequently, the proportion of patients with stage III or IV disease increased from 66% at first consultation to 74% at the initiation of radiotherapy. Among 151 patients treated with curative intent, only 34 (22.5%) of the patients received concurrent chemotherapy while the reaming patients received radiotherapy alone. The 5-year overall survival rate was 28.4% (20.5% in the worst-case scenario). As expected, survival was lower in patients with advanced stage at initiation of radiotherapy and in those treated as palliative care.

**Conclusion:**

The survival of cervical cancer patients remains low in Ethiopia because of late presentation and delay in receipt of radiotherapy, leading to stage migration in substantial proportion of the cases. Concerted and coordinated multisectoral efforts are needed to promote early presentation of cervical cancer and to shorten the unacceptable, long waiting time for radiotherapy.

## Background

Cervical cancer is the fourth most common type of cancer among women worldwide [1, 2]. Of the 500,000 cervical cancer cases and 270,000 deaths estimated to occur worldwide each year, 85% of them occur in LMICs, which are least equipped to screen and treat these patients because of poor healthcare infrastructure [2, 3]. The burden is highest in Eastern Africa, with estimated age-standardized incidence rates of > 30 per 100,000 women [3, 4]. In this region cervical cancer remains a leading cause of cancer death in women, accounting for one in five of all cancer deaths [5].

Ethiopia with the population of more than 110 million, is among countries with the highest incidence rate of cervical cancer worldwide, with about 6000 new cases diagnosed each year [6, 7]. Cervical cancer is the second most commonly diagnosed and the second leading cause of cancer death in the country, with age standardized incidence and mortality rate of 26.4 and 18.4 per 100,000 population each year, respectively (GLOBOCAN 2012) [8]. One of the major challenge of cervical cancer care is Ethiopia is delay in diagnosis, since most of the patients seek medical care at advanced stage of the disease [7, 9]. Tikur Anbessa Specialized Hospital is the only oncology center for the radiotherapy treatment, which is the major treatment modality for locally advanced and advanced cervical cancer; all patients are referred to this hospital from all over the country [9, 10]. However, the number of cervical patients is continuously increasing from year to year, which makes the waiting time for receipt of radiation treatment to be very long [7, 9, 10]. Therefore, delays in treatment is another major challenge of cervical cancer care in Ethiopia.

## Methods

### Study setting and treatment modality

The study was carried out in adult oncology department of Tikur Anbesa Specialized Hospital (TASH) radiotherapy center, under the Addis Ababa University. During the study period in 2014 (January 1, 2014 to December 31, 2014), there were three clinical oncologists, six residents, one General practitioner, and one palliative care physician serving the center. The center had two cobalt-60 radiotherapy machines. The center had no brachytherapy machine during the study period.

Patients with cervical cancer were referred from all over Ethiopia to Tikur Anbessa Hospital Radiotherapy center for radiotherapy. Up on first oncologist consultation and/or radiotherapy planning, tumor stage were classified according to the International Federation of Gynecology and Obstetrics (FIGO) staging system [11]. The stage was based mainly on clinical pelvic examination by at least one clinical oncologist from radiotherapy center. The routine radiological examination such as chest X-ray and abdomino-pelvic ultrasound examination will aid to classify the FIGO stage furthermore, in case of hydronephrosis or metastatic lesion was detected.

According to the treatment protocol of the center, cervical cancer patients FIGO stage IIA and lower were treated surgically with radical hysterectomy and pelvic lymphadenectomy (Wertheim). Patients with FIGO stage IIB and above without surgery or patients who undergone surgery but has positive surgical margins and positive lymph nodes required radiotherapy. Patients with early stage disease but unable to undergo surgery due to other medical conditions also treated with radiotherapy. The radiotherapy planning techniques were two-dimensional (2D-technique). Generally, patients with early stage IIIB and/or stage less than early IIIB with good general condition were treated with curative dose radiotherapy in two phases. However, for patients with FIGO stage above late stage IIIB and/or for patients in emergency state palliative radiotherapy is usually offered. The radiation dose and fractionation depend on the intent of treatment, with curative intent cases treated with conventional RT with radiation dose of 66–72 Gys in two phases while palliative intent cases treated with hypofractionation with varied radiation doses which could be 10 Gys single fraction, 20 Gys in 5 fractions, 30 Gys in 10 fractions based on disease stage and general health status of the patient (e.g., state of renal failure or uremia and presence or absence of Fistula).

### Study design and statistical analysis

Women with histologically verified Cervical cancer (International Classification of Disease-Oncology (ICD-O-3) codes C53.0–9) who were treated in 2014 at the Radiotherapy Center at Tikur Anbessa Hospital Addis Ababa, Ethiopia, were included in the study, retrospectively.

Information on demographic and tumor characteristics, treatments, and response to treatment at 1 year was extracted from the patients’ medical record files. The date of diagnosis was assigned as the date of referral with the suspicion of cervical cancer or the date of biopsy confirmation, whichever is first. Information on vital status was obtained from medical records or the patients or –in case of death- from the relatives through telephone calls. If patients or relatives were not reached by telephone, the last date of personal contact in Tikur Anbessa hospital was taken from the patients’ files. For these patients the additional worst-case scenario analysis was performed with assumption of dead after 6 months of last follow-up date registered on their medical record [12] . The telephone calls were made between 01/02/2018 to 30/03/2018.

The primary end point of this study was overall survival. Groups are compared using parametric (t test) and nonparametric (Chi-square, Mann-Whitney U test) tests. Kaplan-Meier curve and survival analysis was performed using Log-rank test to assess significant differences between groups regarding survival. All *p* values less than 0.05 were considered significant. All data were analyzed using SPSS 20.0 (IBM Corp. Released 2011. IBM SPSS Statistics for Windows, Version 20.0. Armonk, NY: IBM Corp.) and R 3.4.2. (R Core Team (2017). R: A language and environment for statistical computing. R Foundation for Statistical Computing, Vienna, Austria. URL https://www.R-project.org/.)

The ethical clearance was obtained from the Ethical review committee of the Radiotherapy unit of Tikur Anbessa Hospital under Addis Ababa University College of Health Sciences (AAU CHS). Informed consent was waived by the same ethics committee that approved the study, considering the study was mainly based on the retrospective record analysis.

## Result

### Characteristics of the patients and their treatment

Three hundred forty-nine cervical cancer patients received radiotherapy in Black Lion Hospital in 2014. Of those, 242 patient files could be retrieved. The median age at diagnosis was 48 years (range 27 to 86). The average number of births they gave was 5.62 (±2.88). HIV was relatively the commonest comorbidity, in which 26 (11%) were found to be positive (Table 1).

**Table 1:** Basic demographic data and treatment modality

	N (%)
Marital status	
Married	222 (92%)
Divorced	6 (2%)
Widowed	14 (6%)
Histology	
Adeno-squamous	2 (1%)
Adenocarcinoma	7 (3%)
Squamous Cell Carcinoma	233 (96%)
Comorbidity	
Hypertension	14 (6%)
HIV	24 (10%)
Diabetes mellites	3 (1%)
Breast cancer	1 (< 1%)
Address	
Oromia	84 (35%)
Addis Ababa	61 (25%)
Amhara	63 (26%)
SNNPR	18 (7%)
Tigray	14 (6%)
Somali	2 (1%)
Prior surgery	26 (11%)
Prior Chemo	7 (3%)
Stage at First Oncol. Consult.	
I/II^a^	82 (34%)
III	108 (45%)
IV	52 (21%)
Stage at Start RT	
I/II^b^	64 (26%)
III	122 (50%)
IV	56 (23%)
Stage migration	31 (13%)
Concurrent chemo	34 (14%)
Intent of RT	
Curative	151 (62%)
Palliative	91 (38%)

^a^Among 82 patients with stage I/II, 75 (91%) patients was FIGO stage IIB, 5 (6%) was stage IIA and 2 (3%) was stage IB

^b^Among 64 patients with stage I/II, 61 (95%) patients was FIGO stage IIB, 2 (3%) were stage IB and 1 (2%) was stage IIA

Most of the patients had no surgery for cervical cancer prior to their presentation to radiotherapy center; only 26 (11%) patients underwent surgery. The median waiting time for radiotherapy was 5.6 months (range 2 to 9 months). During the waiting time, 31 (13%) patients showed stage migration from their baseline at the first oncologist consultation. There was a difference in waiting time for initiation of RT based on the stage and intent of treatment. About 55 patients (60.4%) s treated with palliative intent received RT within 3 months after diagnosis, while only 31 patients (20.5%) received RT within 3 months in curative intent groups (*p* < 0.001). The waiting time for receipt of RT also differed by stage. The number of patients who received RT with 3 months were 13 (15.1%), 38 (44.2%) and 35 (40.7%) for stage I/II, III and IV, respectively (*p* < 0.001).

Thirty-four (14%) patients received concurrent chemotherapy with radiation while the remaining (majority of) patients received radiation therapy alone. Most of the patients were had lost from follow-up in Tikur Anbessa hospital before 1 year (Table 1).

### Survival

Among 242 patients included in the study, 140 (57.9%) were known dead while 102 (42.1%) patients were alive or censored. For worst-care scenario we estimated that patients had died 6 months after their last follow up if they were not responding on telephone calls. This results in 184 (76%) deceased, while 58 (24%) were known alive within 6 months before the study closure date.

The estimated 60 months (5 year) overall survival probabilities were 28.4% (SE 4.0) in the main analysis. The one-year, two-year, three-year and four-year overall survival probabilities were 77% (SE 2.8), 45.3% (3.5), 36.0% (SE 3.4) and 32.2% (SE 3.5), respectively. The median overall survival time was 23.0 months (95% CI 18.5–27.5).

However, in the worst-case scenario the 60 months (5 year) overall survival probability reduced to 20.5% (SE 3.2) and the median overall survival to 18 months (95% CI 15.6–20.3). The one-year, two-year, three-year and four-year overall survival probabilities were 74.3% (SE 2.8), 35.2% (SE 3.1), 27.2% (SE 2.9) and 24.4% (SE 2.9), respectively.

**Fig. 1:**
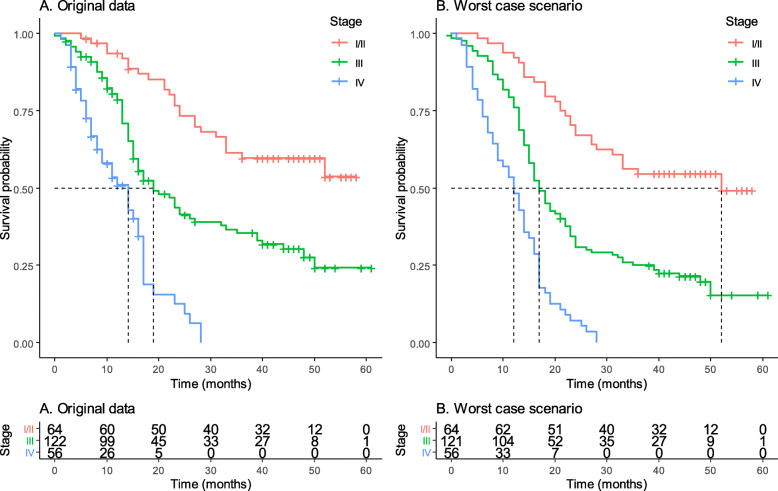
Overall survival of patients according to the stage during receipt of radiotherapy: **A** Kaplan-Meier analysis with original data, and **B** The worst-case scenario Kaplan-Meier analysis

FIGO stage has significant impact on survival of patients. The median of stage I/II is not reached due to survival percentage in this group, 53.7% is above 50% needed for median. Therefore, median survival for stage I/II group is below 53.7% and mean survival is 42.0 (95% CI 38.2–47.6) months. The estimated median time of survival based on the stage at the initiation of radiotherapy was, 19.0 (95% CI 13.4–24.6) and 14.0 (95% CI 9.2–18.8) months for stage III and IV, respectively (*p* < 0.001) in main analysis (Fig. 1).

In the worst-case scenario, the median survival in I/II is reached, but only one patient died and the survival percentage is 49.2% (SE 7.6%). The median survival for stage I/II groups is 52 months, but no confidence interval can be calculated. The mean survival for this group is 40.7 (95% CI 35.9–45.4%) months. The estimated median time of survival based on the stage at the initiation of radiotherapy declined to 17.0 (95% CI 14.7–19.3) and 12.0 (95% CI 9.2–14.7) months for stage III and IV, respectively (*p* < 0.001) in main analysis (Fig. 1).

**Fig. 2:**
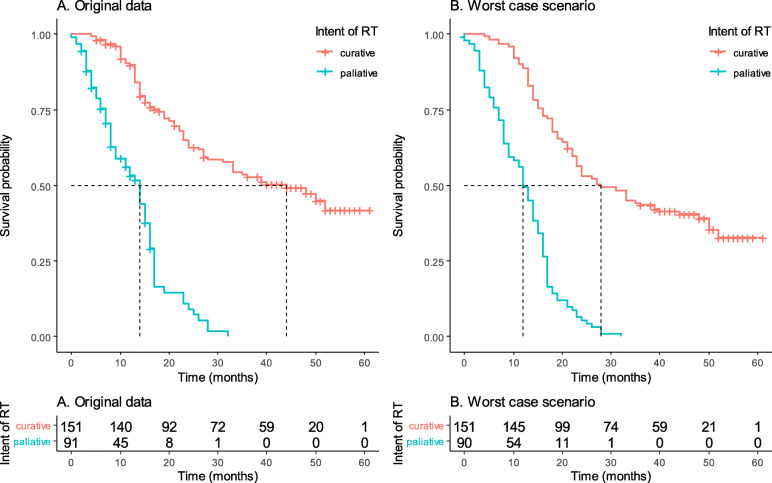
Overall survival of patients according to the intent of radiotherapy: **A** Kaplan-Meier anaysis with original data, and **B** The worst-case scenario Kaplan-Meier analysis

As expected, patients treated with curative intent had significantly better survival as compared to those treated with palliative intent. The median survival was 44.0 (95% CI 31.0–56.9) and 13.0 (95% CI 11.2–16.7) months for patients treated with curative and palliative intent, respectively in main analysis group (*p* < 0.001). In the worst-case scenario the median survival time was 28.0 (95% CI 20.9–35.0) and 12.0 (95% CI 10.3–13.7) months for patients treated as curative and palliative intent, respectively (*p* < 0.001) (Fig. 2).

## Discussion

To our knowledge, this study is the first study to report on the 5-year survival of cervical cancer patients in Ethiopia. The 1-year and 2-year overall survival rates in our study (77 and 45.3%) were substantially lower than those reported in previous studies in TAHRC. Kantelhardt et al. reported a one-year and two-year survival rates of 90 and 74% (45% in the worst-cases analysis), respectively [12]. Similarly, Moelle and colleagues et al. documented a one-year and two- year survival of 84% (54% in the worst case) and 64% (35% in the worst case), respectively, based on patients treated between 2008 to 2013 [13]. While the clinical characteristics and treatment patterns of patients between our study and those previous studies are generally similar, waiting time for initiation of radiotherapy after first consultation with oncologist was substantially longer in our study (5.8 months vs. 3.8 months) [12]. This may in part have contributed to the lower survival rates in our study due to greater disease progression and stage migration. Since our study was conducted 2018 compared to the other two studies in 2013, there were more patients actively using a mobile phones and thus better information on the endpoint (death) could be obtained. Since survival probabilities of our study are within the range of the worst-case analysis of the other two studies, we assume to have ascertained more deaths in our main analysis leading to the lower survival probability in the main analysis.

In general, our survival findings are 15–25% lower than those reported in high income countries such as the United States and in some countries in Africa [14] in part because of lack of standard of care such as brachytherapy, known to improve cervical cancer survival [15, 16]. In a single institution experience from Ghana, patients treated with Coblt-60 machine and then with brachytherapy boost has 86% of 3-year overall survival, [17] which is much higher than our findings in patients treated with curative intent radiotherapy which is 50%. In contrast, stage-specific cervical cancer survival rates in Kenya where patients are treated only with Cobalt-60 machine, reported median survival of 18, 15 and 11 months for Stage II, III and IV patients, respectively [5]; and in our cases 40.7, 17 and 12 months for stage I/II, stage III and stage IV patients, respectively. Here, the median survival for stage II patients were higher in our case, but the median survival of stage III and IV is comparable. However, brachytherapy service in TAHRC has been available to cervical cancer patients since 2015. It will be of interest to assess whether survival of cervical cancer patients has improved in the hospital following the availability of this life-saving therapy.

Concomitant chemo-radiotherapy for locally advanced cancer patients has become standard of care since the late 1990s, after several study documented the survival benefit of this therapy compared to radiotherapy alone [18–20]. The treatment protocol of cervical cancer in Tikur Anbessa hospital radiotherapy center also recommends concurrent chemo radiotherapy especially in curative setting [12]. However, only 14% of the patients in our cohort received combined treatment, and these patients had a better survival than those who received radiotherapy alone. Barriers to receipt of concurrent chemotherapy are likely thought to be financial hardship, high patient load, lack of space and lack of coordination [13].

In addition to sub-standard treatments, the low survival rate of cervical cancer patients in our study in part reflects late stage presentation, as is the case in many sub-Saharan African countries [5, 21, 22]. The majority of our patients had stage III or IV disease at the time of first oncologist consultation, which is consistent with previous studies in the country [12, 13]. Most of these patients received palliative radiotherapy, [23] and in shorter waiting time than patients with early-stage disease. However, this may have compromised the survival of patients with early-stage disease, who may benefit more from timely treatment. While expansion of radiotherapy services (number of radiotherapy machines and personnel) is likely to take many years for optimal solution, some novel strategy is needed in the short term for timely treating of patients with early-stage disease, who may benefit more from timely radiotherapy.

A strength of our study is it showed long-term survival probability of cervical cancer and the predictors for survival in Ethiopia. It also showed the impact of longer waiting time on the overall treatment outcome. It can also help as the reference for the assessment of success in various actions taken to improve access to care. Limitations of our study include missing information (medical records) for many patients and lack of regular follow up after radiotherapy. Our findings of survival rates may be affected by loss to follow-up or ascertainment bias. We did not also have data on radiotherapy dose and schedules, adherence to treatments, general health performance, and nutritional status, which all influence survival.

## Conclusion

The survival of cervical cancer patients in Ethiopia (treated in TAHRC) remains low because of late stage presentation, suboptimal treatments (lack of brachytherapy), and disease progression while waiting for radiotherapy. These findings underscore the need for concerted, multisectoral efforts to improve early detection access to timely and high-quality radiotherapy services.

## Data Availability

The datasets used and/or analysed during the current study are available from the corresponding author on reasonable request.

## Abbreviations

LMICs: Low and Middle-Income Countries
RT: Radiotherapy
WHO: World Health Organization
HPV: Human Papilloma Virus
TASH: Tikur Anbesa Specialized Hospital
ICD-O-3: International Classification of Disease for Oncology, Third edition
CR: Complete Response
PR: Partial Response
PD: Progressive disease
SD: Stable Disease
HIV: Human Immuno-Deficiency Virus
Gy: Gray (unit)
